# Genetic diversity and relationship among Indonesian local sheep breeds on Java Island based on mitochondrial cytochrome b gene sequences

**DOI:** 10.1186/s43141-023-00491-z

**Published:** 2023-03-17

**Authors:** Alek Ibrahim, Endang Baliarti, I Gede Suparta Budisatria, Wayan Tunas Artama, Rini Widayanti, Dyah Maharani, Luis Tavares, Endang Tri Margawati

**Affiliations:** 1 Department of Animal Production, Faculty of Animal Science, Universitas Gadjah Mada, Yogyakarta, Indonesia 55281; 2 Research Center for Applied Zoology, National Research and Innovation Agency (BRIN), Bogor, Indonesia 16911; 3 Department of Biochemistry and Molecular Biology, Faculty of Veterinary Medicine, Universitas Gadjah Mada, Yogyakarta, Indonesia 55281; 4 Department of Animal Breeding and Reproduction, Faculty of Animal Science, Universitas Gadjah Mada, Yogyakarta, Indonesia 55281; 5 Department of Animal Science, Faculty of Agriculture, Universidade Nacional Timor Lorosa’e, Dili, Timor-Leste

**Keywords:** Genetic variation, Indonesian local sheep, Maternal origin, mtDNA Cyt b gene, Phylogenetic

## Abstract

**Background:**

The cytochrome b (Cyt b) gene is one of the most studied mitochondrial DNA (mtDNA) genes to determine sheep’s genetic profile. This study aimed to determine the genetic diversity and relationships of several Indonesian local sheep populations on Java Island, Indonesia, based on the mtDNA Cyt b gene sequences. Blood samples were collected from forty-one individual sheep in seven populations of Indonesia local sheep breeds on Java Island (Priangan = 6, Garut = 6, Batur = 7, Wonosobo = 5, Javanese Thin-Tailed/JTT = 7, Javanese Fat-Tailed/JFT = 5, and Sapudi = 5). DNA extraction was performed on blood samples, and the mtDNA Cyt b gene was amplified using specific primers (Alek-CBF: 5'-CAACCCCACCACTTACAA-3' and Alek-CBR: 5'-CCTTGAGTCTTAGGGAGGTT-3'). The polymerase chain reaction (PCR) products were then sequenced, and data were analyzed using the MEGA version 7.0, DNA SP version 6.0, and NTSYS-pc version 2.11 software.

**Results:**

A total of 1140 bp complete mtDNA Cyt b gene sequences in this study obtained 1134 monomorphic sites (I), six polymorphic sites (V), one segregation site (S), and five parsimony informative sites (P) with a nucleotide diversity (Pi), the average number of nucleotide differences (K), and sequence conservation (SC) were 0.00119, 1.35610, and 0.9947, respectively. There were six haplotypes consisting of two unique haplotypes and four shared haplotypes with a haplotype diversity (Hd) of 0.5415. The genetic distance within and between populations ranged from 0.0000 to 0.0016 and 0.0000 to 0.0020, respectively. Wonosobo, JFT, and Sapudi sheep have the closest relationship, and then these three breeds were close to JTT sheep, followed by Batur, Priangan, and Garut sheep. Two haplogroups have been found based on the Ovine haplogroup clustering. All Wonosobo, JTT, JFT, Sapudi sheep, and most Batur sheep were clustered into haplogroup B. In contrast, Garut sheep were mostly clustered into haplogroup A, while Priangan sheep were clustered into both haplogroups with the same percentage.

**Conclusion:**

Seven Indonesian local sheep breeds on Java Island have a close relationship clustered into two haplogroups, namely haplogroups A and B. Most Indonesian local sheep breeds on Java Island in this study were clustered into haplogroup B, except for Garut and Priangan sheep.

## Background

Domestic sheep (*Ovis aries*) have an essential role in diverse human societies as a source of food, hide, wool, income, and a role in religious and cultural activities. In Indonesia, sheep are exceedingly popular among farmers, especially smallholder farmers, because they are easy to maintain and low cost. Indonesian local sheep breeds have been developed by the community for many years, have adapted to the environment of certain regions, and have historical value in certain areas in Indonesia [1–3]. Until 2022, eleven Indonesian local sheep breeds have been recognized by the Ministry of Agriculture of the Republic of Indonesia, spread across various regions in Indonesia [4]. The Indonesian local sheep breeds’ names are usually based on the source development areas of its local sheep, for example, Garut sheep from Garut Regency, Wonosobo sheep from Wonosobo Regency, Sapudi sheep from Sapudi Island, Batur sheep from Batur district-Banjarnegara Regency, Sakub sheep from Sakub hill-Brebes Regency, Palu sheep from Palu Regency, or Kisar sheep from Kisar Island. The Ministry of Agricultural of Indonesia also develops local sheep breeds such as Compass Agrinak sheep. Indonesian local sheep breeds originating from Java Island are Priangan, Garut, Sakub, Batur, and Wonosobo sheep. In addition, other local sheep thrive on this island, namely Sapudi, Javanese Fat-Tailed (JFT), and Javanese Thin-Tailed (JTT) sheep [4, 5].

Mitochondrial DNA (mtDNA) has been the most popular marker of molecular diversity in animals over the last three decades. Most mtDNA investigations focused mainly on the Cytochrome b (Cyt b) and control region [6, 7]. Cyt b is mtDNA gene that contains abundant phylogenetic information among interspecies and intraspecies. It is considered a good marker for studying the genetic diversity and phylogenetic relationships among species within the same genus and family [7]. This gene is a member of protein-coding genes with a high evolutionary rate and higher variation ratio than other functional genes [8]. The mtDNA Cyt b is widely used for genetic diversity and phylogenetic relationship determination in domestic animals, such as goats [9–12], chickens [13–15], ducks [16, 17], pigs [18], cattle [8, 19, 20], horses [14, 21], buffaloes [22, 23], and sheep [6, 24–27].

Information on the genetic diversity of native and local livestock in Indonesia is important in developing breeding and conservation strategies [8]. Indonesian local sheep, including those on Java Island, are local livestock resources that need to be studied for their genetic profile. In a previous study using mtDNA D-loop on several Indonesian local sheep breeds on Java Island, two haplogroups were found, namely haplogroups A and B [28]. In-depth studies still need to be done to determine the genetic diversity, phylogenetic relationship, and origin of the sheep using various techniques and gene targets. This study aimed to determine the genetic diversity and relationships of several Indonesian local sheep populations on Java Island based on the mtDNA Cyt b gene sequences.

## Methods

### Ethical approval

This study has been authorized and approved with the Ethical Clearance Certificate of the Faculty of Veterinary Medicine Research Ethics Commission, Universitas Gadjah Mada, with approval number 002/EC-FKH/Int./2019. The approval was also obtained from the National Political and Unity of Yogyakarta Province with the approval number 074/1850/Kesbangpol/2019 and from the Agricultural Officers and the National Political and Unity Officers in each of the sample areas used.

### Sample collection

**Fig. 1:**
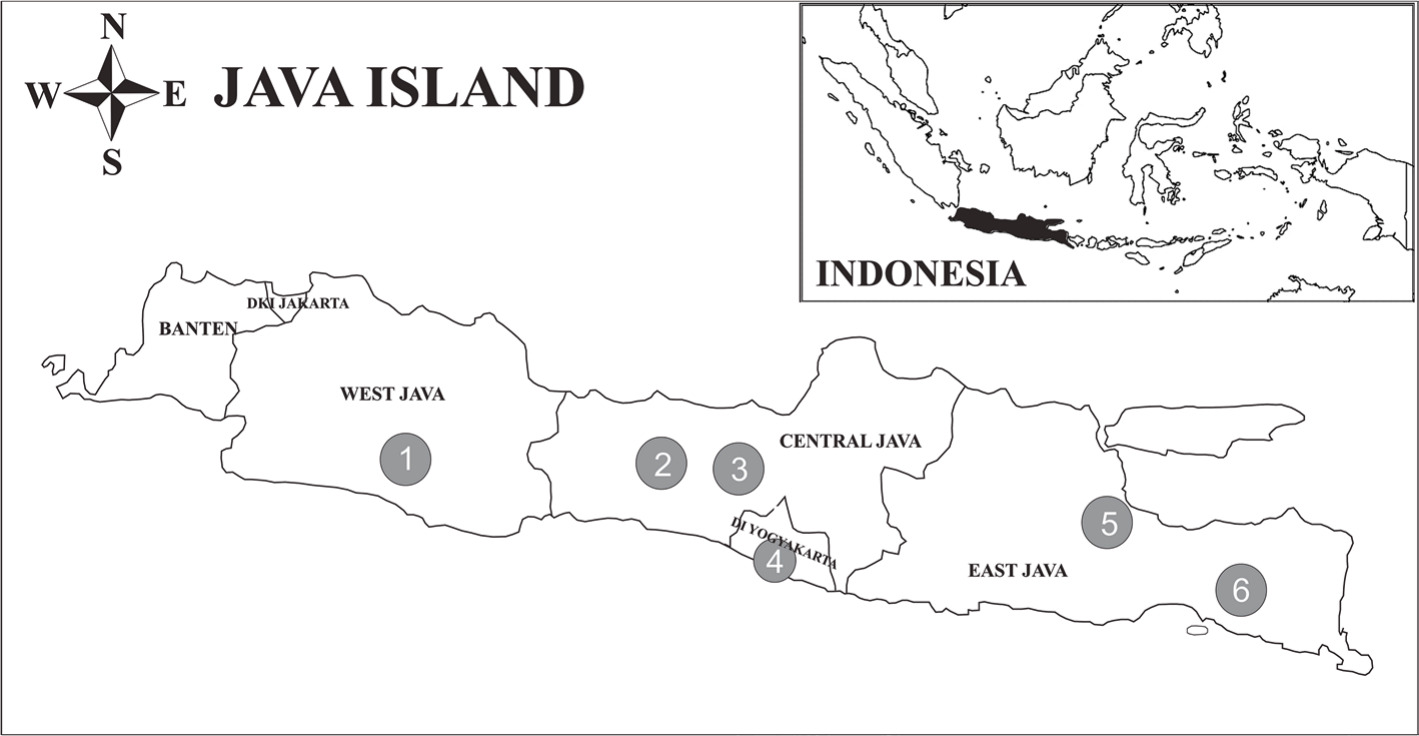
Sampling sites Indonesian local sheep breeds on Java Island, Indonesia: 1. Garut Regency (Garut sheep and Priangan sheep); 2. Banjarnegara Regency (Batur sheep); 3. Wonosobo Regency (Wonosobo sheep); 4. Bantul Regency (Javanese Thin-Tailed sheep); 5. Pasuruan Regency (Javanese Fat-Tailed sheep); 6. Jember Regency (Sapudi sheep)

This study was conducted using blood samples from forty-one individual sheep in seven populations of Indonesian local sheep breeds on Java Island, Indonesia, as presented in Fig. 1. Sheep were sampled by a purposive sampling method, namely by determining each local sheep breed's origin and development areas, and then determining the sub-districts and villages for sampling. Blood samples were taken using a 3-cc syringe through the jugular vein previously cleaned with alcohol. The blood samples were then collected in vacutainer tubes with an anticoagulant (ethylenediaminetetraacetic acid), stored in a cooler box containing an ice pack, and transported to the laboratory for further analysis. The samples were collected from April 2019 to March 2021. However, this study was conducted from October to December 2022 in the Laboratory of Biochemistry and Molecular Biology, Faculty of Veterinary Medicine, Universitas Gadjah Mada.

### Molecular techniques

**Fig. 2:**
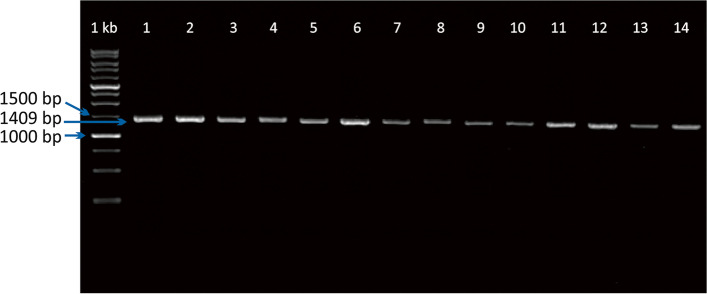
Visualization of PCR product on the targeted of mtDNA Cyt b on Wonosobo sheep (1–4), Batur sheep (5–7), Javanese Thin-Tailed sheep (8–11), and Garut sheep (12–14)

The DNA was extracted based on the manufacturer’s standard protocol using PureLink™ Genomic DNA Mini Kits (Invitrogen). The mtDNA Cyt b gene was amplified directly from the genomic DNA by polymerase chain reaction (PCR). The primers were designed using the Primer3 online version 4.1.0 program (http://primer3.UT.ee/) [29] based on the data from the mitochondrial genome of *Ovis aries* (GenBank accession number: AF010406.1). The mtDNA Cyt b primer sequences were Alek-CBF: 5′-CAACCCCACCACTTACAA-3′ and Alek-CBR: 5′-CCTTGAGTCTTAGGGAGGTT-3′, generated 1409 bp of the PCR product [5]. The PCR reaction consisted of 2 µL of DNA template, 25 µL of KAPA2G Fast Ready Mix + Dye (Kapa Biosystems Ltd.), 2 µL of forward primer, 2 µL of reverse primer, and 19 µL of ddH_2_O. The PCR amplification was conducted using Cleaver® GTC96S (Cleaver Scientific Ltd.) according to the program: 5 min of pre-denaturation at 94 °C, followed by 35 cycles, each consisting of denaturation at 94 °C for 30 s, primers annealing at 55 °C for 40 s, extension at 72 °C for 90 s, then ending with a final extension at 72 °C for 8 min, and storage at 4 °C. The PCR product was visualized using 1.5% agarose gel, and electrophoresis was run at 80 mV for 45 min. The result of amplification could be seen on the ultraviolet illuminator. The purified PCR products were sequenced by 1st BASE-Asia, Malaysia. A request was made to perform a gel extraction at the ordering stage. An example of PCR product visualization results is presented in Fig. 2.

### Data analyses

The primers used in this study can amplify 1409 bp of PCR products and flank complete sequences in the targeted mtDNA Cyt b gene (1140 bp). The sequence products were analyzed using the Molecular Evolutionary Genetics Analysis (MEGA) version 7.0 software [30]. The reference of mtDNA Cyt b gene sequences of wild and domestic sheep of known haplogroup (HapG) types [31] was downloaded from GenBank (https://www.ncbi.nlm.nih.gov) and used as comparators of Indonesian local sheep breeds on Java Island to determine the genetic relationship and assume their origin (Table 1). The mtDNA Cyt b sequences were aligned using Clustal W [32] in the MEGA version 7.0 software. The genetic variation analysis was obtained based on the nucleotide differences of the mtDNA Cyt b sequences. The genetic distance analysis was obtained with the Kimura-2-parameters methods [33]. The phylogenetic tree (UPGMA) was constructed with the 1000 × bootstrap [34] and Kimura-2-parameter method [33]. The genetic diversity and haplotype diversity values were analyzed using DNA Sequence Polymorphism (DNA SP) version 6.0 software (Universitat de Barcelona, Spain) [35]. The phylogenetic tree between Indonesian local sheep breeds was constructed using NTSYS-pc version 2.11 software [20] based on the genetic distance values between breeds.

**Table 1:** References of domestic and wild sheep

Group membership	GenBank accession number
*Ovis aries*	
Haplogroup A	HM236174, HM236175
Haplogroup B	HM236176, HM236177
Haplogroup C	HM236178, HM236179
Haplogroup D	HM236180, HM236181
Haplogroup E	HM236182, HM236183
*Ovis musimon*	HM236184
*Ovis vignei*	HM236187
*Ovis ammon*	HM236188
*Ovis orientalis*	KF312238
*Ovis canadensis*	MH094035
*Ovis nivicola*	MH779626
*Ovis dalli*	MH779627

## Results

### Sequence variation and genetic diversity

The 1140 bp of complete mtDNA Cyt b sequence resulted based on the alignments of the PCR product sequences and the reference sequences. The average percentage of the nucleotide composition of the Indonesian local sheep breeds’ mtDNA Cyt b gene sequences is presented in Table 2. Wonosobo, JTT, JFT, and Sapudi sheep have the same percentage of thymine composition (27.02%), and the higher was found in Garut sheep (27.15%). Wonosobo, JFT, and Sapudi sheep have the same percentage of cytosine composition (28.60%), and the lower was found in Garut sheep (28.46%). The higher percentage of adenine composition was found in Garut sheep (31.46%), and the lower was found in Wonosobo, JTT, JFT, and Sapudi sheep with the same percentage (31.40%). In addition, a higher percentage of guanine composition was found in JTT sheep (13.02%), while the lower was found in Garut sheep (12.92%).

**Table 2:** The average percentage (%) of nucleotide composition in Indonesian local sheep breeds based on mtDNA Cyt b gene sequences

Breed	T(U)	C	A	G	C + G
Priangan	27.11	28.51	31.45	12.94	41.45
Garut	27.15	28.46	31.46	12.92	41.39
Batur	27.09	28.52	31.42	12.97	41.49
Wonosobo	27.02	28.60	31.40	12.98	41.58
JTT	27.02	28.56	31.40	13.02	41.58
JFT	27.02	28.60	31.40	12.98	41.58
Sapudi	27.02	28.60	31.40	12.98	41.58
Overall	27.06	28.55	31.42	12.97	41.52

*T* thymine, *C* cytosine, *A* adenine, *G* guanine, *U* uracil

**Fig. 3:**
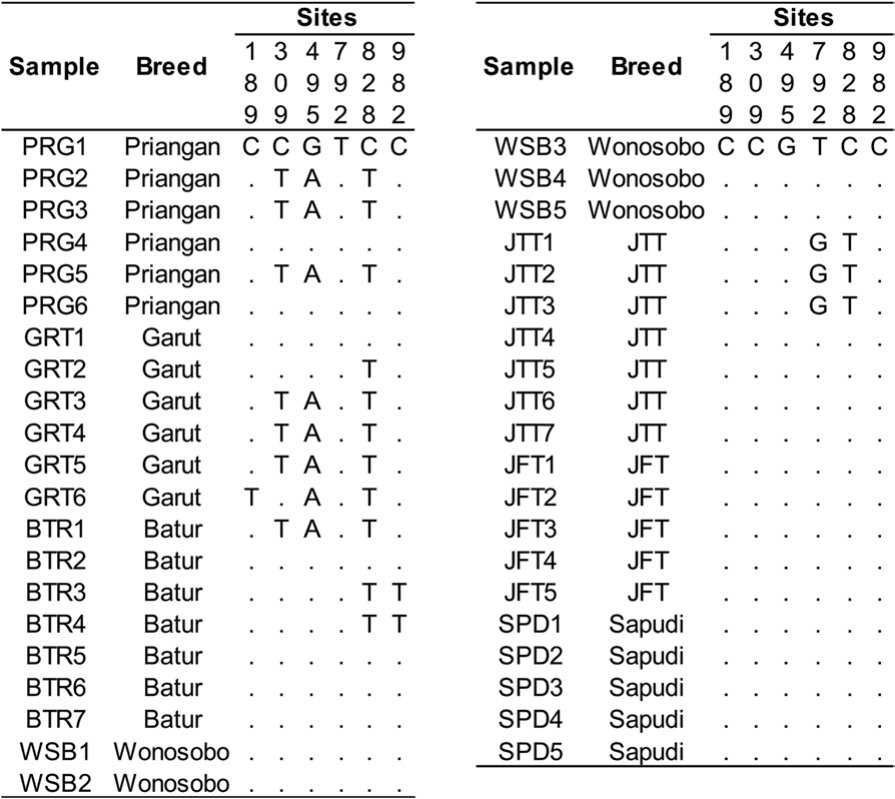
Variable sites position of mtDNA Cyt b gene sequences in Indonesian local sheep breeds

The mtDNA Cyt b gene sequences were aligned in polymorphism sites (variable sites), as presented in Fig. 3. It is shown that the complete mtDNA Cyt b sequence (1140 bp) in the individual sample was only found in six different nucleotide sites between observed samples, namely at the 189th, 309th, 495th, 792nd, 828th, and 982nd sites. The genetic diversity parameters of the mtDNA Cyt b gene sequence of seven Indonesian local sheep breeds on Java Island are presented in Table 3. This table shows that from the forty-one samples used with the 1140 bp of complete mtDNA Cyt b sequence was found 1334 monomorphic sites (I), six variable sites (V), one segregation site (S), and five parsimony informative sites (P) with the nucleotide diversity (Pi), the average number of pairwise differences (K), and sequence conservation (SC) were 0.00119, 1.35610, and 0.9947, respectively. Not found the insertion and deletion in the sequence analysis.

**Table 3:** Nucleotide diversity in Indonesian local sheep breeds based on mtDNA Cyt b gene sequences

Breed	*n*	*I*	*V*	*S*	*P*	Indel	SC	Pi	K
Priangan	6	1137	3	0	3	0	0.9974	0.00158	1.80000
Garut	6	1136	4	2	2	0	0.9965	0.00158	1.80000
Batur	7	1136	4	2	2	0	0.9965	0.00142	1.61905
Wonosobo	5	1140	0	0	0	0	1.0000	0.00000	0.00000
JTT	7	1138	2	0	2	0	0.9983	0.00100	1.14286
JFT	5	1140	0	0	0	0	1.0000	0.00000	0.00000
Sapudi	5	1140	0	0	0	0	1.0000	0.00000	0.00000
Overall	41	1134	6	1	5	0	0.9947	0.00119	1.35610

*n* number of samples, *I* number of monomorphic sites (invariable sites), *V* number of polymorphism sites (variable sites), *S* number of segregation sites (singleton sites), *P* parsimony-informative sites, *Indel* insertion-deletion, *SC* sequence conservation, *Pi* nucleotide diversity, *K* average number of pairwise differences

The haplotype diversity parameters of seven Indonesian local sheep breeds based on mtDNA Cyt b gene sequences were presented in Table 4. It is shown that based on analysis of the sample used was found six haplotypes (Hap) consisting of two unique haplotypes (Hap-3 and Hap-4) and four shared haplotypes (Hap-1, Hap-2, Hap-5, and Hap-6) with the haplotype diversity (Hd) value was 0.5415. The distribution of each haplotype is presented in Table 5. It is shown that haplotype 1 (Hap-1) has the most haplotype members, they were 27 members consisting of three Priangan sheep, one Garut sheep, four Batur sheep, five Wonosobo sheep, four JTT sheep, five JFT sheep, and five Sapudi sheep members. In haplotype 2 (Hap-2), seven sample members consisted of three Priangan sheep, three Garut sheep, and one Batur sheep. In addition, haplotype 3 (Hap-3) and haplotype 4 (Hap-4) each have one sample member, while Hap-5 and Hap-6 have two and three sample members, respectively.

**Table 4:** Haplotype diversity in Indonesian local sheep breeds based on mtDNA Cyt b gene sequences

Breed	*n*	nHap	Hd	nHapG A	nHapG B
Priangan	6	2	0.600	3	3
Garut	6	4	0.800	4	2
Batur	7	3	0.667	1	6
Wonosobo	5	1	0.000	0	5
JTT	7	2	0.571	0	7
JFT	5	1	0.000	0	5
Sapudi	5	1	0.000	0	5
Overall	41	6	0.541	8	33

*n* numbers of samples, *nHap* numbers of haplotype, *Hd* haplotype diversity, *nHapG A* numbers of haplogroup A, *nHapG B* numbers of haplogroup B

**Table 5:** Haplotypes distributions in Indonesian local sheep breeds based on mtDNA Cyt b gene sequences

Haplotypes	Breed							Overall	
	PRG	GRT	BTR	WSB	JTT	JFT	SPD	*n*	%
Hap-1	3	1	4	5	4	5	5	27	68.85
Hap-2	3	3	1	–	–	–	–	7	17.07
Hap-3	–	1	–	–	–	–	–	1	2.44
Hap-4	–	1	–	–	–	–	–	1	2.44
Hap-5	–	–	2	–	–	–	–	2	4.88
Hap-6	–	–	–	–	3	–	–	3	7.32
Total	6	6	7	5	7	5	5	41	100.00

*Hap* haplotype, *n* number of samples, *PRG* Priangan sheep, *GRT* Garut sheep, *BTR* Batur sheep, *WSB* Wonosobo sheep, *JTT* Javanese Thin-Tailed sheep, *JFT* Javanese Fat Tailed sheep, *SPD* Sapudi sheep

**Fig. 4:**
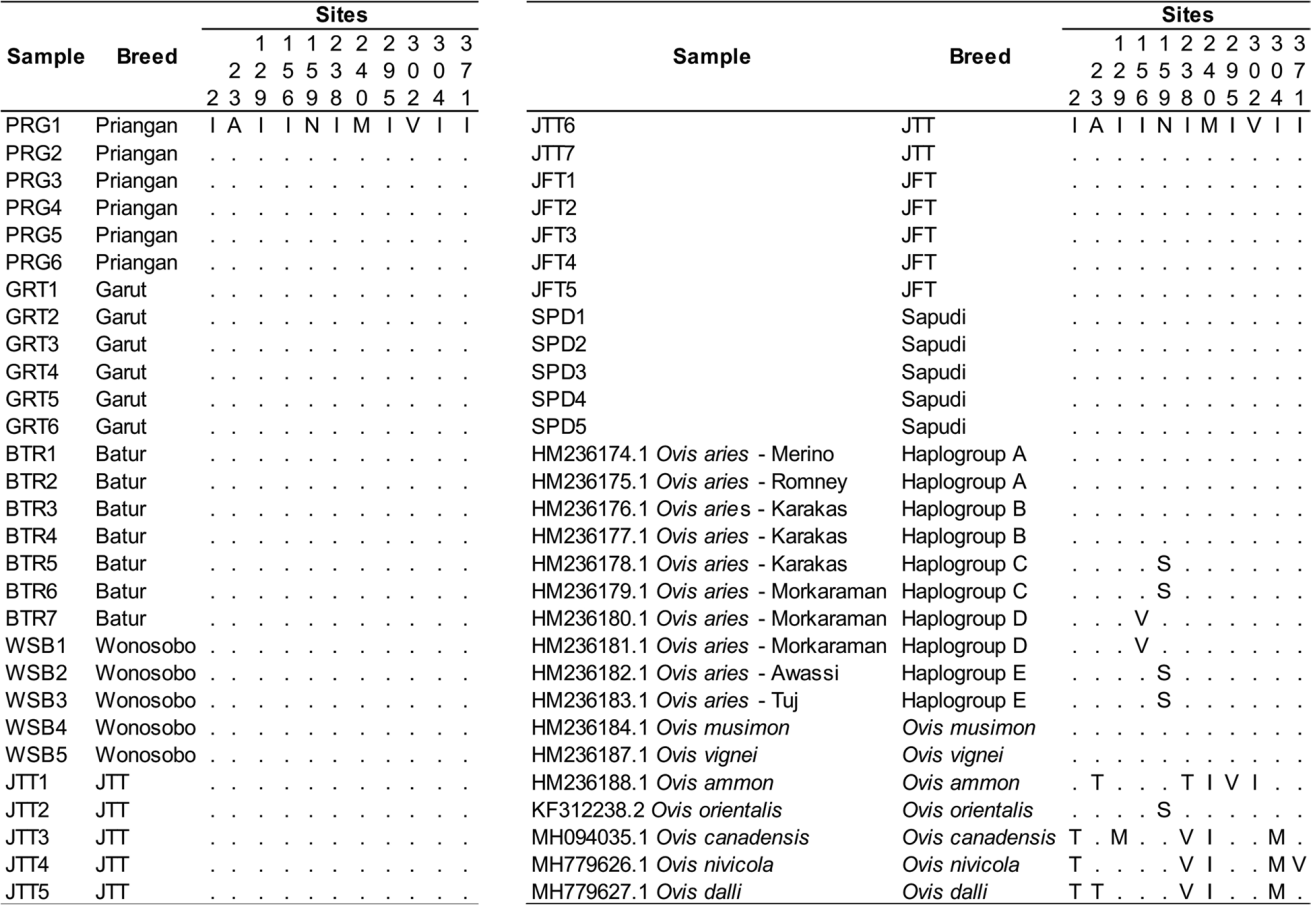
Amino acid diversity of mtDNA Cyt b gene in Indonesian local sheep breeds

This study’s alignment of amino acids was conducted by comparing the sample sequences to the *Ovis aries* sequences from various haplogroups and genera (Table 1). Alignment of 1140 bp complete mtDNA Cyt b sequence in this study was found 380 amino acids. There were 11 different amino acids from several amino acids obtained compared to the references, but there was no difference in amino acids between Indonesian local sheep breeds in this study (Fig. 4).

### Genetic distance of Indonesian local sheep breeds and *Ovis aries* haplogroups

**Fig. 5:**
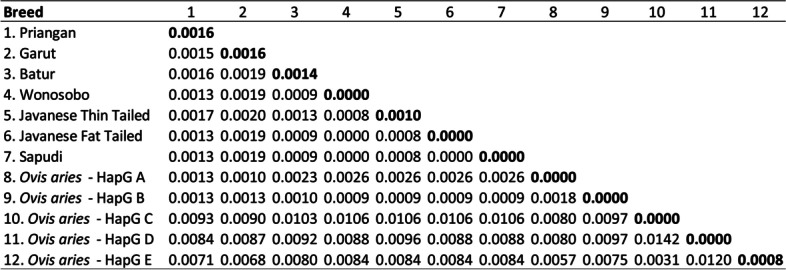
Genetic distance within (bold font) and between (standard font) breeds of Indonesian local sheep breeds based on mtDNA Cyt b gene sequences

The genetic distance between seven Indonesian local sheep breeds on Java Island and the sheep reference in the study is presented in Fig. 5. It is shown that genetic distance within the population (0.0000–0.0016) and between populations (0.0000–0.0020) in this study were categorized to close distance. The farthest distance value within the population was found in Priangan and Garut sheep with the same value (0.0016), while the closest distance value was found in Wonosobo, JFT, and Sapudi sheep (0.0000). In addition, the farthest distance value between the population was found between Garut and JTT sheep (0.0020), while the closest distance value was found between Wonosobo and JFT sheep, Wonosobo and Sapudi sheep, and JFT and Sapudi sheep with the same values (0.0000).

### Phylogenetic analysis

**Fig. 6:**
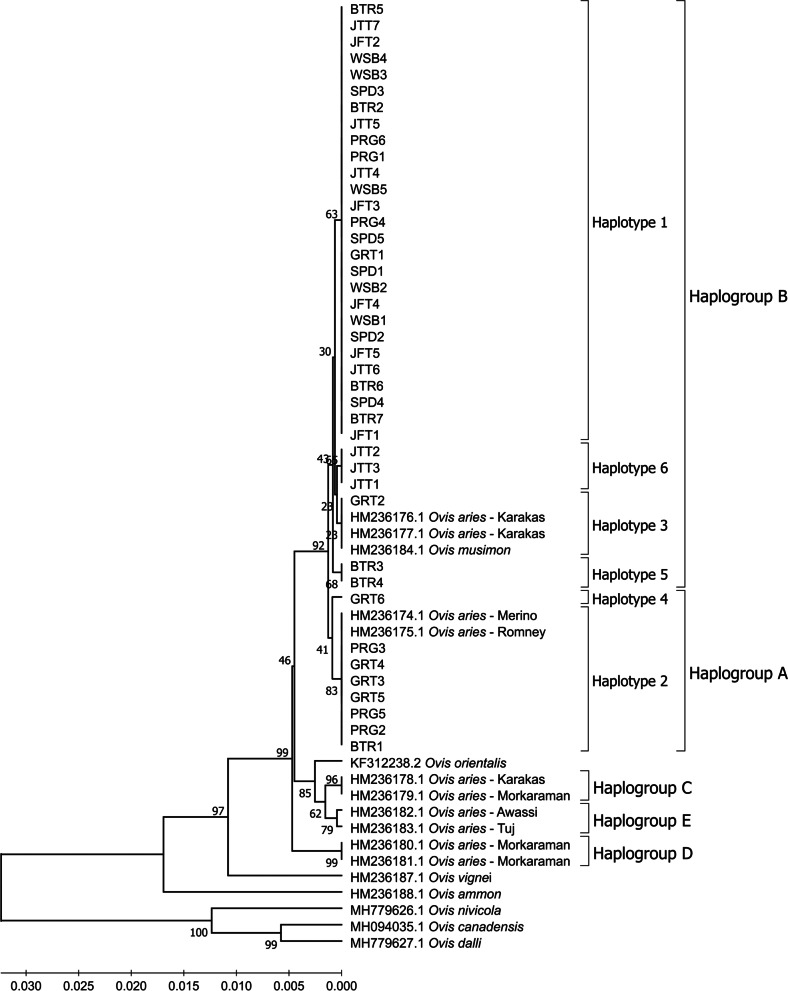
Phylogenetic tree of Indonesian local sheep breeds and the other sheep in various genera and haplogroups based on mtDNA Cyt b sequences. PRG: Priangan sheep, GRT: Garut sheep, BTR: Batur sheep, WSB: Wonosobo sheep, JTT: Javanese Thin-Tailed sheep, JFT: Javanese Fat Tailed sheep, and SPD: Sapudi sheep

**Fig. 7:**
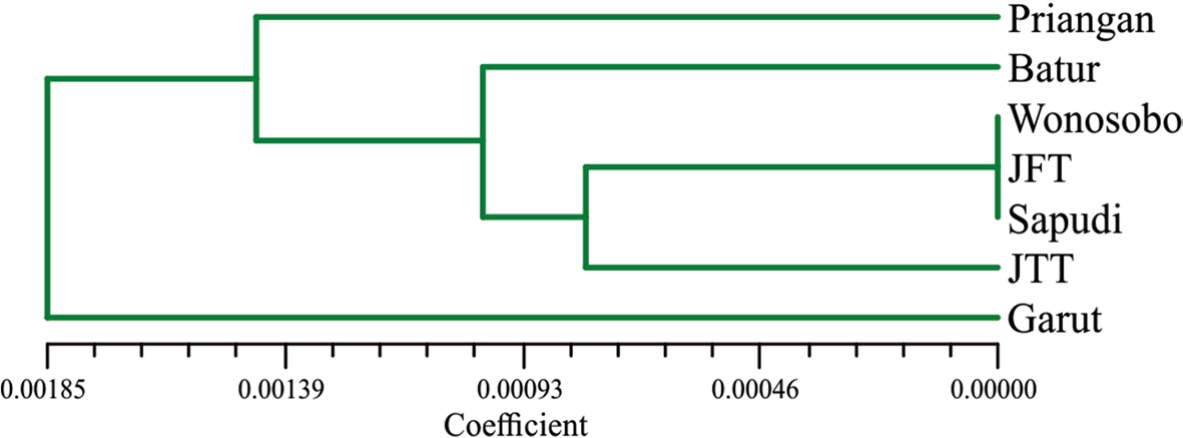
Phylogenetic tree of observed Indonesian local sheep breeds based on genetic distance values of mtDNA Cyt b gene sequences

The phylogenetic tree of Indonesian local sheep breeds and the sheep breeds referenced in this study are presented in Fig. 6. The phylogenetic analysis resulted that Indonesian local sheep breeds on Java Island were clustered in two haplogroups, namely haplogroup A (HapG A) and haplogroup B (HapG B). Haplogroup A consists of haplotypes 2 and 4, while haplogroup B consists of haplotypes 1, 3, 5, and 6. In this study, the phylogenetic tree between Indonesian local sheep breeds on Java Island was constructed based on the genetic distance between them, as presented in Fig. 7.

## Discussion

The present results show that the nucleotide base composition of the mtDNA Cyt b gene on all observed Indonesian local sheep has almost the same percentage. Overall, nucleotide base composition from the highest to lowers, namely adenine (31.42%), cytosine (28.55%), thymine/uracil (27.06%), and guanine (12.97%). The Wonosobo, JTT, JFT, and Sapudi sheep have the same percentage of G + C (41.58%) and have a higher value than the other sheep observed, while the lower value was the Garut sheep (41.39%). Overall, the G + C percentage (41.52%) in observed Indonesian local sheep breeds was lower than the A + T percentage (58.48%). The nucleotide composition in this study was similar to published studies in goats [36], chicken [37], and cattle [7] that the G + C nucleotides composition in these animals was lower than A + T nucleotides composition.

Our results show that nucleotide diversity within and between breeds was only found in Priangan, Garut, Batur, and JTT sheep. The Wonosobo, JFT, and Sapudi sheep have the same mtDNA Cyt b gene sequence. This figure also shows that Priangan and Garut sheep were the sheep breeds with the highest nucleotide variation. No unique nucleotide base sequence was found in the different sheep breeds observed in this study. It indicates that no specific nucleotide base in the mtDNA Cyt b gene sequence can be used to characterize Indonesian local sheep breeds on Java Island in this study.

Furthermore, the results show that Priangan and Garut sheep have a higher nucleotide diversity/Pi (0.00158) and the average number of pairwise differences/K (1.8000) than other observed sheep breeds. Batur sheep had slightly higher nucleotide diversity than JTT sheep. In the same number of samples (7 samples), Batur sheep had slightly more variable sites than JTT sheep, so the values of Pi (0.00142 vs 0.00100) and K (1.61905 vs 1.4286) were also higher, and lower sequence conservation (SC) values (99.65% vs 99.83%). Wonosobo, JFT, and Sapudi sheep were monomorphic, or there was no difference in nucleotide sequences in the mtDNA Cyt b gene. The low Pi value was also found in nineteen local sheep breeds in Xinjiang (0.00052–00,665) [38] and six Egyptian sheep breeds (0.00087–0.00595) [6]. The low average pairwise differences (K) value were also found in Rahmani sheep (0.982), Saidi sheep (1.735), and Fallahi sheep (2.066) [6]. Based on within breeds, the higher haplotype diversity (Hd) was found in Garut sheep (0.800) from six samples used, resulting in four haplotypes, while the lower value was found in Wonosobo, JFT, and Sapudi sheep (0.000), resulting in the same haplotype in each breed (Tables 4 and 5). The Hd value in the Xinjiang local sheep [38], Mongolian native sheep [39], and Egyptian sheep [6] breeds were 0.464–0.893, 0.800–0.960, and 0.643–0.871, respectively.

The present results show that in 41 samples of observed Indonesian local sheep no difference in amino acid, both within and between sheep breeds. In this study, the nucleotide base changes occurred on the third codon, except for the C > T nucleotide base change that occurred in the first codon of the 982nd site (Fig. 3). Differences in nucleotide sequences do not change the amino acid structures of the mtDNA Cyt b gene in observed Indonesian local sheep, and the amino acid structure remains the same in all observed Indonesian local sheep breeds. It indicates that the mtDNA Cyt b gene was conserved relatively and that most nucleotide mutations do not change the amino acid structure [26]. This study’s absence of amino acid diversity indicates that the mtDNA Cyt b gene cannot be used as a unique marker to characterize the observed Indonesian local sheep breeds on Java Island. Nucleotide mutation can be occurred by transition mutation and transversion mutation. Nucleotide mutation in the protein-encoding genes can be resulting synonymous amino acids (silent substitution) and non-synonymous amino acids. Most non-synonymous amino acids are due to the substitution of nucleotides in the first and second codons [11]. This study has no difference in amino acid structure due to the observed sheep breeds clustered into *Ovis aries* of haplogroup A and/or haplogroup B (Fig. 4). These haplogroups have the same amino acid structure in the mtDNA Cyt b gene. The difference in structure occurred when compared to *Ovis aries* of haplogroups C, D, and E in the 156th site (I > V) and 159th site (N > S). The mtDNA Cyt b gene sequence of Indonesian local sheep breeds in this study also has the same amino acid structure as *Ovis musimon* and *Ovis vignei* (Fig. 4).

Based on the genetic distance value, Indonesian local sheep breeds in this study have a close relationship with domestic sheep from haplogroup A and haplogroup B (Fig. 5). Priangan sheep have the same genetic distance value between *Ovis aries* from haplogroups A and B (0.0013). The genetic distance between Garut sheep and Haplogroup A sheep (0.0010) was lower than between Garut sheep and haplogroup B sheep (0.0013). It indicates that Garut sheep are closer to domestic sheep clustered in haplogroup A. In contrast, the genetic distance in Batur, Wonosobo, JTT, JFT, and Sapudi sheep was higher between domestic sheep in haplogroup B (0.0023–0.0028) than in haplogroup A (0.0009–0.0010). It indicates that Batur, Wonosobo, JTT, JFT, and Sapudi sheep were closer to domestic sheep clustered in haplogroup B than haplogroup A. These results reflect a previous study that Indonesian local sheep breeds clustered to haplogroup A and haplogroup B. They were mostly closer to haplogroup B than haplogroup A based on mtDNA D-loop sequences [28, 40]. The genetic distance in this study was lower than the Northernmost snow sheep population (0.0020–0.0028) [41] and six Egyptian sheep breeds (0.0012–0.0053) [6].

The phylogenetic analysis reported that the previous study clustered the domestic sheep into five haplogroups, namely haplogroups A, B, C, D, and E in Asia and Europe sheep breeds [40]. Indonesian local sheep in this study were clustered into haplogroup A and haplogroup B (Fig. 6). All Wonosobo, JTT, JFT, and Sapudi sheep were clustered into haplogroup B. Most Batur sheep were also clustered into haplogroup B, except for BTR1 sheep clustered into haplogroup A. Garut sheep were clustered into haplogroup A, except for GRT1 and GRT2. In addition, Priangan sheep have the same number of members clustered into both haplogroups. Thus, most of the Indonesian local sheep breeds on Java Island in this study were clustered into haplogroup B, except for Garut and Priangan sheep. These results were consistent with previous studies using mtDNA D-loop sequences that Indonesian local sheep breeds can be clustered into two haplogroups, namely haplogroup A and haplogroup B [28, 40]. Predominantly haplogroup B than haplogroup A was also found in Altanbulag sheep [1], Romanian Racha sheep [42], Tsigai sheep and Cikta sheep [27], but in contrast to Nepal sheep [43], Mongolian sheep [1, 39], and Tibetan sheep [44].

Our results indicate that Wonosobo, JFT, and Sapudi sheep have the closest relationship. These three sheep breeds were then close to JTT sheep, followed by Batur, Priangan, and Garut sheep. This study found that most Garut sheep were clustered into haplogroup A, while Priangan sheep have the same percentage in haplogroups A and B (Table 4 and Fig. 6). This study also found one sample of Batur sheep clustered into haplogroup A. The researchers in the previous study stated that haplogroup A in the domestic sheep is predominantly found in Asian breed types [25, 33], while haplogroup B is predominantly found in European breed types and partially in Eastern Asia breed types [40, 45–48]. This study indicates that most Indonesian local sheep breeds on Java Island originated from European breed types. It is also supported by the close relationship between Indonesian local sheep breeds and *Ovis musimon* (European Mouflon) in the phylogenetic analysis (Fig. 6). It contrasts with Meadows et al. [40] and Guangxin et al. [49] report that domestic sheep from Indonesia, Mongolia, and Tibet have a close relationship with Asian sheep or domestic sheep with haplogroup A, while domestic sheep with haplogroup B are predominantly found in European sheep. It can be an exception for domestic sheep breeds in Indonesia, considering that Indonesia is a country with a strategic area for exploring and trading with various countries. In addition, this country had previously been occupied by the Dutch government for more than 300 years [50]. During that time, various livestock was imported from outside the area to be developed in this country including sheep [51]. Thus, Indonesian local sheep breeds may have resulted from crosses with breeds of European origin [28, 40, 48, 52].

The Garut, Priangan, and Batur sheep which were partly clustered into haplogroup A could be due to the crossing with Merino sheep in these three sheep, or maybe these sheep were indeed of an Asian breed of origin [28]. According to the Ministry of Agriculture of the Republic of Indonesia, Garut sheep resulted from crossing between Merino sheep from Australia, Kaapstad sheep from Africa, and Javanese Thin-Tailed sheep (local sheep) [53]. Priangan sheep resulted from crossing between local sheep, Texel sheep, and Merino sheep [54]. Batur sheep resulted from crossing between Merino sheep and thin-tailed sheep (local sheep) [55, 56]. However, genetically it has not been clearly explained in those Ministry of Agriculture Decree. This study proved that Garut, Priangan, and Batur sheep have a close relationship with Merino sheep from Australia (HM236174), clustered with haplogroup A.

This study shows that most Indonesian local sheep breeds on Java Island were clustered to haplogroup B, which these sheep have a close relationship with European sheep breeds, whereas Indonesia on the Asia continental. However, this study also found the sheep samples clustered to haplogroup A, in which these sheep have a close relationship with Asian sheep breeds. Considering the history and facts in this study, the Indonesian local sheep breeds that developed on Java Island were indicated as resulting from crosses between Asian sheep types and European sheep types. This finding corroborates previous studies conducted with mtDNA D-loop sequences [28]. Further research still needs to be done to corroborate these results. Research with more target genes, larger samples, wider sampling area, complex methods, analyses, and comparative data with more various breeds both from Indonesia and outside the country will assist in characterizing and exploring the origins of Indonesian sheep breeds.

## Conclusion

In conclusion, based on the mtDNA Cyt b gene sequence, there was a close relationship between Indonesian local sheep breeds on Java Island. Wonosobo, JFT, and Sapudi sheep had the closest relationship, and then these three breeds were close to JTT sheep, followed by Batur, Priangan, and Garut sheep. All Wonosobo, JTT, JFT, and Sapudi sheep and most Batur sheep were clustered to haplogroup B, which has a close relationship to European breed types. In contrast, most Garut sheep were clustered to haplogroup A, which has a close relationship to Asian breed types. In addition, Priangan sheep were clustered into both haplogroups with the same proportion.

## Data Availability

All data are primary data and generated from the research, research materials belong to our laboratory (Laboratory of Biochemistry and Molecular Biology).

## Abbreviations

A: Adenine
bp: Base pair
BTR: Batur
C: Cytosine
Cyt b: Cytochrome b
D-loop: Displacement loop
DNA: Deoxyribonucleic acid
DNA SP: DNA Sequence Polymorphism
G: Guanine
GRT: Garut
Hap: Haplotype
HapG: Haplogroup
Hd: Haplotype diversity
I: Invariable sites
Indel: Insertion-deletion
JFT: Javanese Fat Tailed
JTT: Javanese Thin Tailed
K: Average number of pairwise differences
MEGA: Molecular Evolutionary Genetics Analysis
mtDNA: Mitochondrial deoxyribonucleic acid
NTSYS: Numerical Taxonomy and Multivariate Analysis System
P: Parsimony-informative sites
PCR: Polymerase chain reaction
Pi: Nucleotide diversity
PRG: Priangan
S: Singleton sites
SC: Sequence conservation
SPD: Sapudi
T: Thymine
U: Uracil
UPGMA: Unweighted Pair Group Method with Arithmetic Mean
V: Variable sites
WSB: Wonosobo
